# Building Otto: An open-source Franz diffusion cell autosampler for automating in vitro skin permeation studies

**DOI:** 10.1016/j.ohx.2025.e00735

**Published:** 2025-12-21

**Authors:** Keng Wooi Ng, Liam Archbold, Wing Man Lau

**Affiliations:** a School of Pharmacy, Newcastle University, King George VI Building, Queen Victoria Road, Newcastle upon Tyne NE1 7RU, United Kingdom; b Translational and Clinical Research Institute, Faculty of Medical Sciences, Newcastle University, William Leech Building, Framlington Place, Newcastle upon Tyne NE2 4HH, United Kingdom

**Keywords:** DRUG absorption, High performance liquid chromatography (HPLC), Automation, Robotics, 3D printing

## Abstract

The Franz diffusion cell (FDC), widely used for measuring drug absorption across the skin, is usually operated manually. However, manual operation is not only labour-intensive and time-consuming, but inevitably introduces human errors and inter-operator variability. The requirement to perform regular sampling around the clock also presents a significant logistical challenge for researchers. Commercial FDC automation solutions are costly and require proprietary/bespoke FDC designs. To overcome these challenges, we have developed Otto as a customisable and affordable, aftermarket FDC automation solution, to be retrofitted to existing FDCs of generic specifications. Otto uses a modified cartesian 3D-printer as a gantry and adds liquid-handling capabilities using 3D-printed components and common, inexpensive laboratory consumables. Liquid samples are collected into standard autosampler vials. Capable of handling 100 samples per run, Otto supports a high throughput and integrates well with downstream analytical equipment, without modifying the FDC or the analytical equipment. Its programming is facilitated by OttoMate, a companion software application with a graphical user interface designed to generate human-readable code for Otto. Here, we describe the design, construction, operation and characterisation of Otto. To our knowledge, this is the first open-source, retrofittable FDC autosampler with such throughput.

## Graphical abstract

**Figure:**
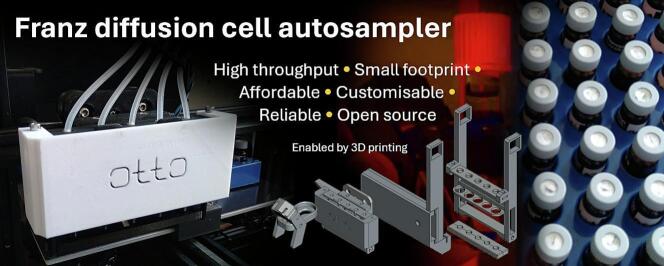

Specifications table

**Table:** 

Hardware name	Otto, the Franz diffusion cell autosampler
Subject area	Medical (Pharmaceutical Science)
Hardware type	Other (liquid handling)
Closest commercial analog	Vision Microette, System 913, Phoenix RDS
Open source license	CC BY 4.0
Cost of hardware	Under 500 British Pounds
Source file repository	https://doi.org/10.17632/cvc9vxjgn9.2
OSHWA certification UID *(OPTIONAL)*	N/A

## 1 Hardware in context

The Franz diffusion cell (FDC) is a standard laboratory apparatus used for the in vitro evaluation of chemical absorption across the skin [1], in line with various regulatory guidelines and recommendations, including the Organisation for Economic Co-operation and Development (OECD) [2], [3], the European Medicines Agency (EMA) [4] and the United States Food and Drug Administration (USFDA) [5]. It is an important tool for product development, environmental protection and regulatory compliance.

Typically made of glass, the FDC consists of two main compartments, i.e., the upper donor chamber and the lower receptor chamber. The skin specimen is sandwiched between these chambers. The drug formulation or substance of interest is introduced into the donor chamber and allowed to diffuse across the skin into the receptor chamber. The receptor chamber is filled with a receptor fluid, which is constantly stirred to ensure homogeneity. A sampling port extending from the receptor chamber allows the receptor fluid to be sampled for analysis, and the receptor chamber to be refilled at defined time intervals, without dismantling the FDC. The temperature of the FDC is usually maintained at 32 ± 1 °C during use, using a water bath, a dry bath or a water jacket.

The FDC is usually operated manually. Although technologically straightforward and cost-effective, this is labour-intensive and time-consuming. Experiments often last between 24 and 72 h, requiring around-the-clock sampling and refilling. Manual operation inevitably introduces human error and inter-operator variability. It also requires complex scheduling to enable sampling during unsociable hours, which entails social, economic, health and safety implications. To avoid sampling during unsociable hours, researchers may pause sampling for an extended duration, but such large sampling gaps could compromise data quality. For example, we have shown previously that an overnight (≥16-hour) pause in FDC sampling can cause excessive drug accumulation in the receptor chamber, violating sink condition and leading to underestimation of drug flux [6].

A FDC autosampler enables around-the-clock, unattended operation without the drawbacks of manual operation. However, current commercial offerings are costly. They also require proprietary/bespoke FDCs so cannot be retrofitted to existing FDCs of different specifications [7], [8], [9]. To overcome these limitations, we have built Otto, a FDC autosampler robot that can be retrofitted to existing FDCs to automate FDC sampling and refilling [6]. Otto offers the following advantages compared to current commercial solutions:

• Affordable
• Customisable
• Can be retrofitted to generic FDCs
• Small footprint

## 2 Hardware description

**Fig. 1:**
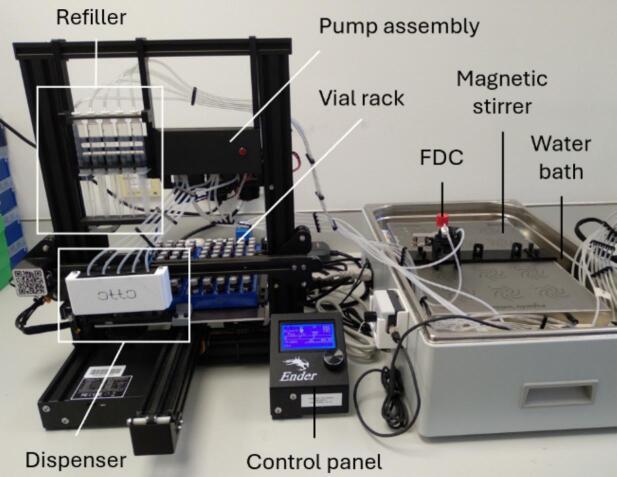
Otto (left) with an exemplar FDC and water bath setup (right).

Otto is adapted from an open-source cartesian 3D printer (Ender 3 Pro, Creality, Shenzhen, China) [10], [11], which serves as the gantry (Fig. 1). The adaptations include electronic components, 3D-printed parts and off-the-shelf consumables. These adaptations are organised into the following core modules/assemblies:

1. Sampler module
2. Refiller module
3. Vial racks
4. Franz diffusion cells

All modifications to the Ender 3 Pro are reversible. Thus, in principle, the 3D-printed parts can be printed on the Ender 3 Pro before converting it into the gantry for Otto. However, access to a separate 3D printer is recommended, as additional parts may need to be printed while assembling Otto (e.g., to replace broken parts). To keep the operating and maintenance costs low, Otto uses minimal moving parts and inexpensive consumables. Standard connectors and snap joints are used to facilitate toolless assembly and disassembly, where possible.

Otto’s design retains the static characteristic of the FDC. Unlike other automation solutions [7], [12], it does not modify the FDC or the downstream analytical equipment. This may encourage its adoption and facilitate regulatory acceptance, as it does not fundamentally alter the way the FDC is used or how the results are interpreted.

To replace manual FDC operation, Otto needs to either solve or circumvent the following workflow challenges:

1. Manoeuvre the sampling and refill needles accurately in and out of the sampling port.
2. Extract an accurate sample volume from the FDC and refill it with an equal volume of fresh receptor fluid consistently across multiple sampling points.
3. Reliably transfer the liquid sample from the FDC into the autosampler vial, and the fresh receptor fluid from the reservoir into the FDC during the refill.
4. Clean or change the liquid handling tools between FDCs, and between sampling and refilling, to avoid cross-contamination.
5. Adhere to the sampling schedule.
6. Handle multiple FDCs simultaneously.

Instead of manoeuvring needles in and out of the sampling port, the sampling and refill needles remain in situ throughout the experiment (circumvents challenge 1). This also removes the need for cleaning or changing the needles between samples. Since each FDC has its own set of sampling and refill needles, there will be no cross-contamination between FDCs (circumvents challenge 4).

Instead of sampling and refilling the FDCs volumetrically, Otto is volume-agnostic and simply samples any liquid within the sampling needle’s reach. The sample volume is determined by the fixed insertion depth of the sampling needle in the sampling port and the volume of receptor fluid refilled into the FDC (solves challenge 2). The sampling needle depth determines the lower meniscus in the sampling port when sampling is complete, before the refill. The refill volume determines the upper meniscus (for a given lower meniscus) after the refill. Fixing both parameters ensures that the sample volume remains consistent across sampling/refilling cycles (see Section 3, FDCSamplingRefill.mp4).

The sampling needle depth is important inasmuch as it is not so low that it introduces air bubbles into the receptor chamber, nor so high that it causes the fluid in the sampling port to overflow after a refill. Provided that these conditions are met, the sample volume depends solely on the refill volume, which is set in OttoMate as the ‘refill step size’ (see Section 6.2). Considering that a 2 mm refill step size produces approximately 240 µL sample volume with the FDCs used in this study, the relationship can be summarised as 120 µL/mm. A peristaltic pump transfers the liquid sample out of the sampling port, through a sampling tube, into an autosampler vial.

To dispense the sample into the sample vial, a dispensing needle, connected to the sampling tube, is inserted by the gantry through the septum in the vial cap. This gantry motion depresses a limit switch, which activates the pump for a pre-programmed duration, sufficient to fully transfer the sample into the vial. The dispensing needle is then retracted from the vial, aided by springs in the dispensing assembly, releasing the limit switch and deactivating the pump in the process. The springs help push the vial down so that it remains in the vial rack (see Section 3, DispenserMechanism.mp4).

In principle, it should be possible to activate sampling using G-code commands (e.g., using extruder commands to control the peristaltic pumps). It is a conscious design decision to use a hardware switch instead to ensure the pumps are only activated if the dispensing needles have successfully penetrated the septa in the vial lids. The limit switch is positioned such that it will not be depressed unless the dispensing needles are in the sample vials. This is to avoid spillage in case the pumps are activated when the needles have not been inserted into the vials successfully. Given that the gantry’s motherboard is located below the vial rack (installed on the build plate), such spillage may present an electrical hazard.

Otto can handle up to 5 FDCs simultaneously (solves challenge 6). The gantry moves the dispenser assembly, which contains 5 dispensing needles, from one row of vials to another between sampling points (solves challenge 3) (see Section 3, OttoOperation.mp4). These tasks are scheduled by pre-programming the gantry movements using G-codes, after which Otto operates fully unattended (solves challenge 5).

FDC refilling is driven by the Z-axis leadscrew, which serves as a syringe pump. The Z-axis motor turns the leadscrew, raising the X-axis to push the syringe plunger by a pre-programmed distance, discharging a fixed volume of liquid from the syringe into the FDC receptor chamber. Since the gantry has only a single leadscrew on the left, the X-axis exhibits more flex on the right, away from the leadscrew. Thus, the syringe pump is positioned on the left, close to the leadscrew. This helps improve volume consistency.

The gantry is programmed using human-readable G-codes. The plain-text G-codes can be viewed and edited in any text editor. However, to avoid human error associated with editing G-codes manually, we have also created the web browser-based OttoMate companion app to generate G-codes based on user input in a graphical user interface. OttoMate is written in HyperText Markup Language (HTML) and cross-browser compatible JavaScript to ensure accessibility and portability. It can be hosted on a static web server or read from local file storage without internet access (Section 6.2).

## 3 Design files summary

Table 1 lists the design files used to complete this build. The fcstd.zip archive contains original computer-aided design (CAD) files in the .fcstd format, created and editable in FreeCAD (https://freecad.org). These were created/last modified in FreeCAD version 1.0.1. The 3mf.zip archive contains the same 3D models in 3D Manufacturing Format (.3mf), ready to slice for 3D printing using suitable slicer software (last tested in PrusaSlicer 2.9.2; https://github.com/prusa3d/PrusaSlicer). The files in these archives are named according to the modules they belong to, described under the build instructions below. For example, components of the sampler module are named ‘sampler-*.fcstd’, components of the refiller module are named ‘refiller-*fcstd’, and components of the pump assembly are named ‘pump-*.fcstd’, etc. Each .3mf file contains the full set of printable parts for the module/assembly, in the required quantities. The individual 3D-printable parts and the number of copies needed of each are shown in the supplementary file, 3D-Printable-Parts-List.pdf.

**Table 1:** Design files for Otto.

Design file name	File type	Open source license	Location of the file
fcstd.zip	CAD files	CC BY 4.0	https://doi.org/10.17632/cvc9vxjgn9.2
3mf.zip	3MF files	CC BY 4.0	https://doi.org/10.17632/cvc9vxjgn9.2
frame-z-limit-switch-bracket.fcstd	CAD file	GPL-3.0	https://doi.org/10.17632/cvc9vxjgn9.2
PTFE-clip-R4-75scaled.stl	STL file	GPL-2.0	https://doi.org/10.17632/cvc9vxjgn9.2
OttoMate.html	HTML	CC BY 4.0	https://doi.org/10.17632/cvc9vxjgn9.2
OttoOperation.mp4	Video	CC BY 4.0	https://doi.org/10.17632/cvc9vxjgn9.2
DispenserMechanism.mp4	Video	CC BY 4.0	https://doi.org/10.17632/cvc9vxjgn9.2
FDCSamplingRefill.mp4	Video	CC BY 4.0	https://doi.org/10.17632/cvc9vxjgn9.2
3D-Printable-Parts-List.pdf	PDF	CC BY 4.0	https://doi.org/10.17632/cvc9vxjgn9.2

The ‘frame-z-limit-switch-bracket.fcstd’ file is a remix of ‘Z limit switch bracket.step’ (available at https://github.com/CrealityOfficial/Ender-3), elongated to shorten the gantry’s travel distance. This is released under the GPL-3.0 licence, in accordance with the upstream licence.

The ‘PTFE-clip-R4-75scaled.stl’ file is ‘PTFE-clip-R4.stl’ (available at https://www.printables.com/model/531604-mmu3-printable-parts/files) downscaled to 75 % in PrusaSlicer version 2.9.2. The original design has not been altered, so no CAD file has been supplied. This is released under GPL-2.0, pursuant to the upstream licence.

OttoMate.html is the browser-based G-code generator for programming Otto’s gantry movements. Its live development repository can be found at https://github.com/ngkengwooi/ottomate.

The video files in .mp4 format show various aspects of Otto’s operation, including the sampling and refilling routine (OttoOperation.mp4), the mechanism of the dispenser assembly (DispenserMechanism.mp4) and an FDC being sampled and refilled by Otto (FDCSamplingRefill.mp4).

These design files have been published on Mendeley Data [13].

## 4 Bill of materials summary

Table 2 shows the bill of materials needed to complete this build.

**Table 2:** Bill of materials for Otto.

Designator	Component	Number	Cost per unit (£)	Total cost (£)	Source of materials	Material type
Gantry	Ender-3 Pro	1	157.79	157.79	Creality	Other
Check valve	Female Luer lock to male Luer lock check valve	1	2.20	11.00	Masterflex # MFLX30505-92	Polymer
Sampling tube	Silicone, 1.0 mm (ID) 3 mm (OD)	1	31.30 (10 m)	12.52	Fisherbrand #FB50853	Polymer
Hose-barb-to-Male-Luer-lock fitting	Male Luer to 1/16-inch hose barb, nylon	15	28.14 (25 pk)	17.05	Masterflex #45505–00	Polymer
Hose-barb-to-Female-Luer-lock fitting	Female Luer to 1/16-inch hose barb, nylon	5	38.05 (25 pk)	7.61	Masterflex #45502–00	Polymer
Peristaltic pump	100 mL/min 12 V DC, 80 mA	3	8.89	26.67	Amazon # B07D7TN1BW	Other
Refill tubing	PVC, 1/16 inch (ID) 1/8 inch (OD)	1	22.00 (100 ft)	5.50	Sigma-Aldrich #Z280348	Polymer
Terminal strip	5A, 6 mm^2^ wires, screw termination	1	5.41	0.54	RS Components #820–8375	Other
Hookup wire red	0.26 mm^2^ 23AWG, PVC insulation	1	15.36 (100 m)	0.31	RS Components #1964305	Other
Hookup wire black	0.26 mm^2^ 23AWG, PVC insulation	1	14.66 (100 m)	0.29	RS Components #196–4298	Other
Equipment wire	Tri Rated, PVC type TI3 insulated, 16 AWG, 30 x 0.25 mm	1	0.89 (1 m)	1.98	Multicomp Pro #PP001272	Other
Limit switch	12 V DC, 2A	1	7.41	7.41	RS Components #682–1929	Other
Syringes	5 mL	5	49.71 (100 pk)	2.49	Fisherbrand #14955458	Polymer
Sampling needle	Stainless steel, 38 mm/22G	5	0.19	0.95	NeedleZ #NB22G1.5	Other
Dispensing needle	Stainless steel, 40 mm/18G	5	170.80 (100 pk)	8.50	BD #303129	Other
Refill needle	PTFE 50 mm/22G	5	1.20	6.00	NeedleZ #BNFLEX22G2	Polymer
Buck converter	24 V DC to 12 V DC, 3A	1	5.00	1.00	Amazon.co.uk #B07DJ5HZ7G	Other
Compression springs	Stainless steel, 29.3 mm x 3.52 mm, 0.16 N/mm	5	1.70	8.50	RS Components #821–273	Metal
Autosampler vial rack	Polypropylene, 50 positions for 1.5 mL autosampler vials	2	26.74	53.48	Fisherbrand #12212187	Polymer
LED Indicator	5 mm 12 V DC red, integrated resistor 680 Ω	1	1.03	1.03	Multicomp # PPOP-120R	Other
3D printing filament	PETG, 1.75 mm diameter, 0.5 kg	1	27.38 (1 kg)	13.69	Multicomp # MC011465	Polymer
M3x6 screws	Hex socket cap, M3 thread, 6 mm length	2	11.65 (50 pk)	0.47	RS Components #280–981	Metal
M3x8 screws	Hex socket cap, M3 thread, 8 mm length	50	14.17 (50 pk)	14.17	RS Components #280–997	Metal
M3x10 screws	Hex socket cap, M3 thread, 10 mm length	10	21.25 (50 pk)	4.25	RS Components #660–4636	Metal
M3x12 screws	Hex socket cap, M3 thread, 12 mm length	10	14.23 (50 pk)	2.85	RS Components #281–007	Metal
M3x40 screws	Hex socket cap, M3 thread, 40 mm length	4	51.18 (200 pk)	1.02	RS Components #822–9060	Metal
M3 square nuts (M3nS)	M3 thread, 5.5 mm width	50	5.30 (100 pk)	2.65	RS Components #837–262	Metal
M3 hex nuts (M3n)	M3 thread, 5.5 mm width	25	5.22 (250 pk)	0.52	RS Components #560–293	Metal

## 5 Build instructions

### 5.1 3D-printable parts

3D-printable parts were modelled using FreeCAD (https://freecad.org). We printed them in polyethylene terephthalate glycol (PETG) using fused deposition modelling, variously on a Prusa i3 MK3.5 and Prusa MINI+ (Prusa Research, Praha, Czechia), although other materials can be used. Earlier prototypes printed in polylactic acid (PLA) also performed well. The parts were sliced in PrusaSlicer 2.7.4 (Prusa Research, Czechia) and printed with a 0.4  mm nozzle at a 0.20  mm layer height, with gyroid infill. Minimal supports were used.

### 5.2 Gantry and frame

3D-printed parts were added to the gantry’s frame to create mounting points for the various liquid-handling components. The pump and refiller assemblies were secured using 3D-printed hangdown brackets hanging down from the top bar of the frame. The stock filament holder was mounted horizontally inside the gantry’s frame to create an additional mounting point for the refiller module, its position defined precisely by the 3D-printed brackets (see Section 5.4). To reposition the filament holder, its screws were loosened, and the filament holder was guided through cutouts in the hang-down mounting brackets. The screws were then tightened to lock it in place.

The home position of the gantry was raised by replacing the Z-axis limit switch bracket with a longer variant. This modification was made to reduce the gantry travel time and thus the overall duration of the sampling/refilling operation.

In principle, it is possible to use other Cartesian 3D printers for the gantry. For example, the Prusa i3 MK3.5 and Prusa MINI+, both cartesian 3D printers, performed the same gantry movements accurately. However, the 3D-printed mounting mechanisms designed for the Ender 3 Pro may not be suitable for other printer frames and may need to be re-designed. Additionally, some printers may have a different home position (e.g., the Prusa MINI+ homes to the front right compared to the Ender 3 Pro’s front left). OttoMate assumes that the home position is on the front left so some hardcoded positions may also need to be changed to optimise G-code generation.

### 5.3 Sampler module

**Fig. 2:**
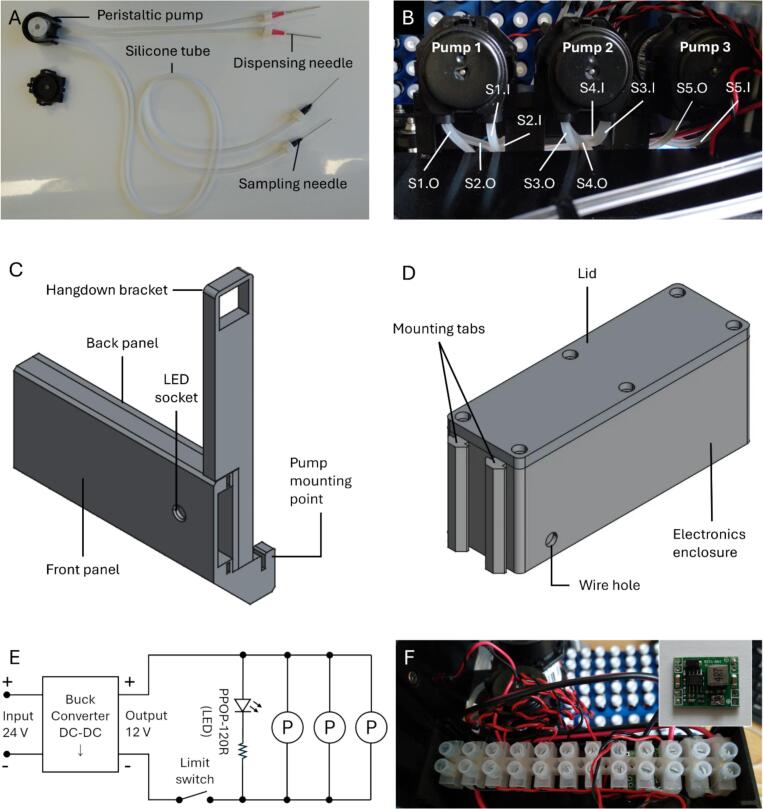
(A) Fluid line for the sampling module. (B) Top view of the peristaltic pumps, mounted on to the frame of the gantry and wired as shown in (E). The sample tubes (labelled S1 for sample tube 1, S2 for sample tube 2, etc.) are shown with the pump outlets (connected to the dispensing needles) denoted as “.O” and the inlets (connected to the FDCs) denoted as “.I”. (C) CAD model of the mounting bracket for the peristaltic pumps. (D) CAD model of the electronics enclosure. (E) Wiring diagram depicting the electronic circuit for operating the peristaltic pumps, which are denoted by the symbol ‘P’. The input voltage was supplied from the motherboard of the gantry. The limit switch was positioned within the dispenser module. The LED was installed in the front panel of the pump assembly, shown in (C). (F) Photograph of the wiring using a terminal strip in the electronics enclosure. The buck converter (inset) was affixed to the bottom of the terminal strip.

The fluid line of the sampler module (i.e., the sampling line) consisted of a set of dispensing needles connected to a set of sampling needles, through some silicone tubes and a peristaltic pump assembly (Fig. 2A). The dispensing needles were housed in a dispenser assembly responsible for activating the peristaltic pumps, dispensing the samples into the autosampler vials, and retracting the dispensing needles from the autosampler vials after sampling.

The gantry must be disconnected from the power source before attempting any electrical component removal or adaptation. A tubing clip (PTFE-clip-R4-75scaled.stl) can be used to organise both the sampling and refill tubes.

#### 5.3.1 Pump assembly

The stock silicone tubes of the pumps were replaced with ones of a smaller diameter (3 mm external, 1.0 mm internal), each 100 cm in length. Two of the three pumps were configured with 2 silicone tubes each, to provide 5 sampling lines in total (Fig. 2B). The silicone tubes were positioned such that the shorter segment (∼30 cm) was the outlet, while the longer segment served as the inlet. 3D-printed duplex tubing clips were fastened onto the tubes and secured to the pumps’ housing, replacing the stock tube clips, to prevent the tubes from slipping during operation. The longer segment of each sampling tube was connected to a sampling needle using a hose-barb-to-male-Luer-lock fitting. The shorter segment was connected to a blunt dispensing needle, also via a hose-barb-to-male-Luer-lock fitting.

During sampling, the gantry lowers to insert the dispensing needles into autosampler vials (Section 5.3.2). Only following successful needle insertion can the gantry reach low enough to depress the limit switch, which activates the pumps and a light-emitting diode (LED) in the front panel. In this design, the limit switch serves as a safety interlock to ensure the pumps do not activate unless the dispensing needles have been securely inserted into the autosampler vials. The duration for which the pump is active is programmed via a G4 dwell command in the G-code. The LED is optional but proved useful for monitoring pump activation and deactivation on video during the development process.

The peristaltic pumps were wired in parallel and mounted on to the frame of the gantry using 3D-printed parts (Fig. 2C). Where necessary, wires were spliced by soldering and insulated with heat-shrink tubing. The electronic components were mostly concealed in a 3D-printed enclosure, mounted on to the frame of the gantry above the power supply unit, by inserting the mounting tabs into the grooves of the aluminium extrusion (Fig. 2D). The power source was the hotend connector in the gantry’s motherboard. A buck converter was used to step down the voltage from 24  V to 12  V for the pumps and LED (Fig. 2E). The hookup wires were used to connect the limit switch, pumps, LED and buck converter via the terminal strip (Fig. 2F). The equipment wire was used to connect the motherboard power source to the buck converter, also via the terminal strip. The wires were inserted through the designated wire holes on either side of the enclosure. With the hotend removed, the pumps (each rated 12  V/80  mA) and LED (12  V/20  mA) would not overload the circuit as their combined power requirement (3.12  W) was lower than the power requirement of the original hotend (40  W).

In earlier prototypes, the pumps were powered using regular alkaline batteries, connected serially in an 8-cell AA battery holder (8 × 1.5 V = 12  V; #185-4633, RS Components, Corby, UK). In that setup, no buck converter was used, but it was necessary to monitor the voltage as the batteries depleted over time. We switched to motherboard power for greater voltage stability, which made troubleshooting easier.

#### 5.3.2 Dispenser assembly

**Fig. 3:**
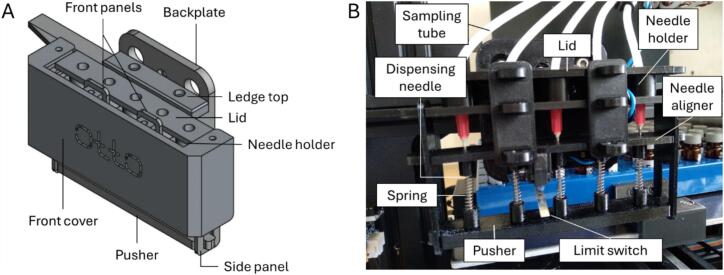
The dispenser assembly. (A) CAD model depicting various 3D-printed parts of the dispenser assembly. (B) Photograph of the dispenser assembly with the front cover removed to reveal the internal mechanisms. The sampling tubes are the peristaltic pump outlets.

The dispenser assembly is shown in Fig. 3. The stock extruder motor and hotend were removed from the X-carriage and unplugged from the motherboard. The backplate was then mounted onto the X-carriage using the original M3 screws for the hotend. A limit switch was attached to the needle guide (needle aligner) using an M3x12 screw and an M3 hex nut (M3n). Two M3 square nuts (M3nS) were inserted into their designated slots in the needle guide. The needle holder and needle guide were inserted into the designated slots in the backplate and secured, along with the ledge top, using two M3x40 screws. The side panels were snapped on and the pusher fitted into the slider slots in the side panels. Five pusher springs were then installed between the needle guide and the pusher, ensuring each spring was seated accurately within its respective pocket at both ends. The silicone sampling tubes were threaded through the holes in the lid and connected to the dispensing needles using hose-barb-to-male-Luer-lock fittings. The dispensing needles were then inserted into their respective openings in the needle holder and needle guide, passing centrally through the springs and the corresponding holes in the pusher. The lid was lowered into position, making sure the rear tabs engaged with the correct slots in the ledge cover, and locked into place with the two front panels. The front cover was then slid horizontally into position, aligning its upper lips over the protruding tabs in the lid. Finally, two M3x40 screws were inserted through matching holes on either side of the assembly to secure it.

### 5.4 Refiller module

**Fig. 4:**
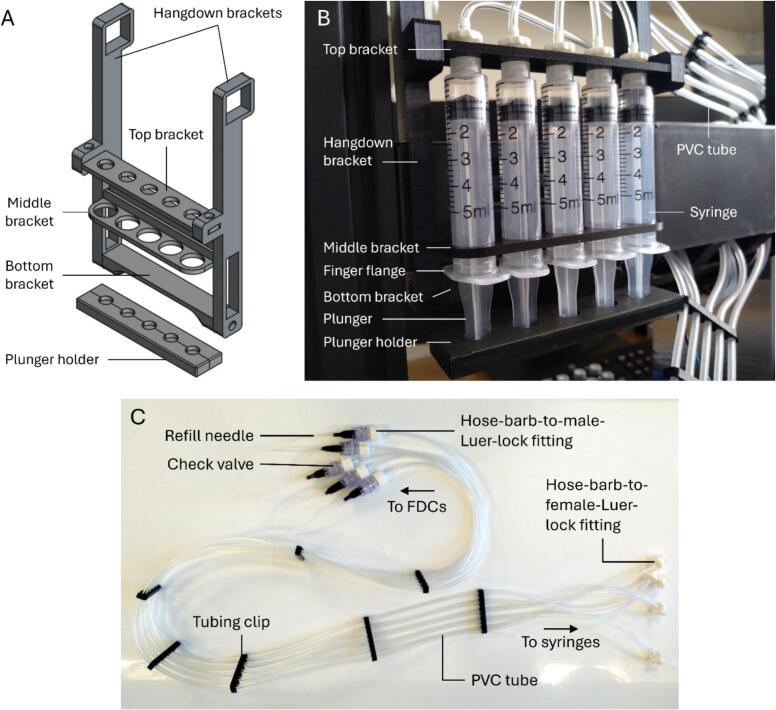
The refiller module. (A) CAD model of the 3D-printed parts. (B) Photograph of the refiller module showing the syringes and fluid lines installed. (C) An overview of the refill fluid line when detached.

The refiller module consisted of 5 parallel 5  mL syringes secured to the frame of the gantry. The top and bottom refiller brackets were affixed to the hang-down brackets using M3x12 screws and M3n nuts. The syringes were inserted into the holes in the middle and top brackets (Fig. 4A–B). The middle bracket was then inserted through the lowest slit in the horizontally mounted filament holder, ensuring one of the finger flanges was inserted into the gap between the filament holder and the bottom bracket. The middle bracket was then secured from the rear with the 3D-printed middle-bracket lock. The parts (a) and (b) of the plunger holder were assembled such that the thumb rests of all five syringes were encased inside them.

The fluid lines for the refiller module (i.e., the refill line) consisted of 5 polyvinyl chloride (PVC) tubes (external diameter: ∼3 mm; internal diameter: ∼1.6 mm), each attached to a syringe in the refiller module via a hose-barb-to-female-Luer-lock fitting on one end, and a check valve attached to the refill needle on the other, also via a hose-barb-to-male-Luer-lock fitting (Fig. 4C). The check valve was included to prevent liquid backflow from the FDC into the refiller module. As such, the check valves must be disconnected when filling the syringes with the receptor fluid. The refill needles were attached to the check valves via a Luer-lock mechanism.

### 5.5 Vial rack

**Fig. 5:**
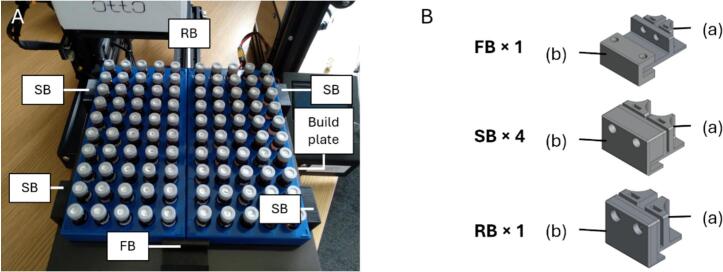
(A) The vial rack, fastened to the build plate using 3D-printed brackets: a front bracket (FB), 4 side brackets (SB) and a rear bracket (RB). (B) Each bracket consisted of 2 parts: part (a) held the vial rack; part (b) attached it to the build plate. Both parts were secured to the vial rack using M3 screws, M3n and M3nS nuts. The round holes were for the screws. FB, SB and RB are not shown to scale relative to each other.

The vial rack was composed of two off-the-shelf 5 × 10 autosampler vial racks, giving a capacity of 100 vials. The combined vial rack was fastened on to the build plate using 3D printed brackets, M3 screws, M3n and M3nS nuts (Fig. 5). The brackets latched on to the edges of the build plate without modifying it. Each bracket consisted of 2 parts: (a) and (b). Part (a) supported the vial rack while part (b) secured it to the build plate. Two M3nS nuts were inserted into the designated slots in the top surface of part (a). Additionally, for the front bracket, two M3n nuts were inserted into the hexagonal pockets under part (a). Two 3.5  mm holes were then drilled into the wall of the vial rack to match the holes in each corresponding bracket. To do this, the wall of the vial rack was inserted into the long groove in part (a). A small hole was initially made using an electric drill with a 1  mm drill bit, through the holes in part (a). This ensured that the holes in the vial rack aligned with those in the bracket. The holes in the vial rack were then enlarged using the electric drill, now fitted with a 3.5  mm drill bit. Part (b) was then slid into place with its bottom lip under the build plate. M3 screws were used to fasten parts (a) and (b) together. The screws used were M3x10 for the side brackets and M3x8 for the rear bracket. The front bracket required M3x12 screws to fasten the vial rack to part (a), and M3x6 screws to fasten part (b) to part (a).

### 5.6 Franz diffusion cell (FDC) assembly

**Fig. 6:**
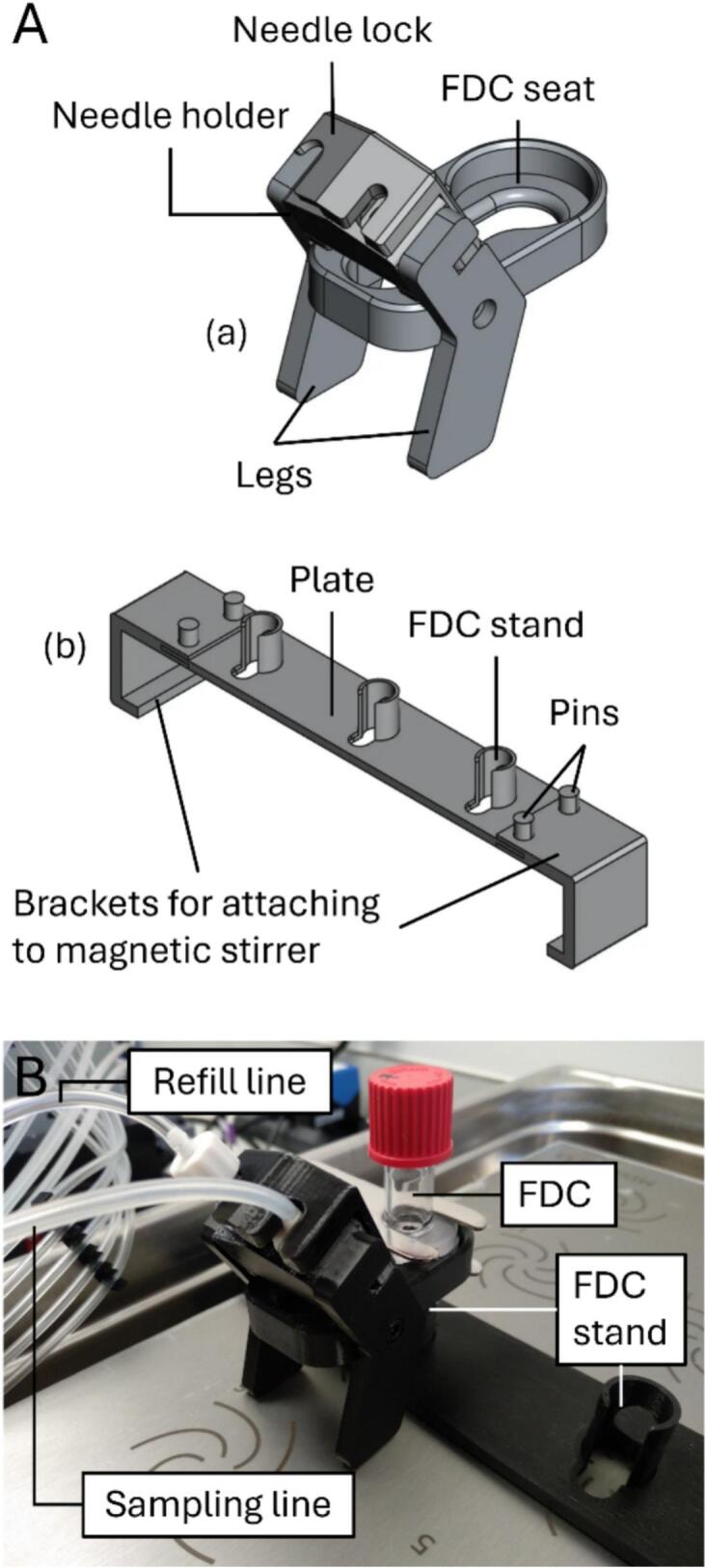
The FDC assembly. (A) CAD models of the 3D printed assemblies for (a) securing the sampling and refill needles to the FDC, and (b) securing the FDC to the magnetic stirrer, aligning them with the stirring positions and keeping them upright. Each FDC stand supports a FDC. Assemblies (a) and (b) are not shown to scale relative to each other. (B) Photograph of a FDC assembly showing the FDC with the sampling and refill needles installed.

The 3D-printed parts were configured for use with 2.5 mL glass FDCs (Cambridge Glassblowing, Ely, UK). They were designed to hold the FDCs upright in a shallow water bath (Clifton™ unstirred digital ShallowBath™, Nickel-Electro, Weston-super-Mare, UK), align them with the stirring positions in the MIXdrive 15 submersible, multi-position, magnetic stirrer (2mag, Munich, Germany), and secured the sampling and refill needles in the sampling port (Fig. 6).

Two M3nS nuts were inserted into the designated slots in the FDC seat. The legs were then secured onto the seat using M3x8 screws. The 3D-printed parts for holding this assembly upright in the water bath (plate, FDC stand, brackets and pins) were snap-fitted and attached to the magnetic stirrer. The FDC was assembled as per manual operation, with the skin/membrane sandwiched between the donor and receptor chambers. The assembled FDC was installed in the FDC seat and secured with a Rotulex™ RC 19 metal clamp (Fisher Scientific, Loughborough, UK). This was then placed in the water bath, held upright by the 3D-printed FDC stand.

## 6 Operation instructions

### 6.1 Calibration

The following calibration steps are aimed at establishing the optimal parameters for the gantry movements and need to be performed before the first use. Recalibration should only be necessary if deviations from these parameters are suspected to impair Otto’s performance.

#### 6.1.1 Axis stability

To ensure the positional accuracy of the gantry, all axes must be stable and the build plate must be level (i.e., be at a constant vertical distance from the dispenser assembly in all positions). Tighten the eccentric nuts holding the axes appropriately. The build plate can be raised, lowered and levelled using the knobs in the four corners under the build plate. We recommend setting the build plate to its lowest position before levelling it.

#### 6.1.2 Clearance

The clearance calibration should be performed to define the optimal axis positions, so that the gantry does not collide with any object during use. First, fill the vial rack with autosampler vials (used but clean autosampler vials may be used) and remove any obstructions around Otto. We used 2 mL SureSTART™ glass vials (Thermo Scientific, Waltham, MA, USA) with pre-slit septa in the caps. Using the ‘Move axis’ command from the control panel, position the dispensing assembly 1–2  mm above the vials. Disable the stepper motors using the ‘Disable steppers’ command from the control panel. Then, manually move the dispenser assembly along both the X- and Y-axes to all four corners of the build plate, as well as diagonally in both directions, while keeping the dispenser assembly consistently 1–2  mm above the vials. Ensure that the Y-axis does not collide with any object in front of or behind Otto, and that no fluid lines are caught by the movements along any axis. If the dispenser assembly collides with the vials, re-level the build-plate (Section 6.1.1). Re-attempt the clearance calibration until satisfactory clearance is achieved in all axes, if necessary. Record the Z-axis position as the ‘needles-up’ position.

#### 6.1.3 Needle-vial alignment

**Fig. 7:**
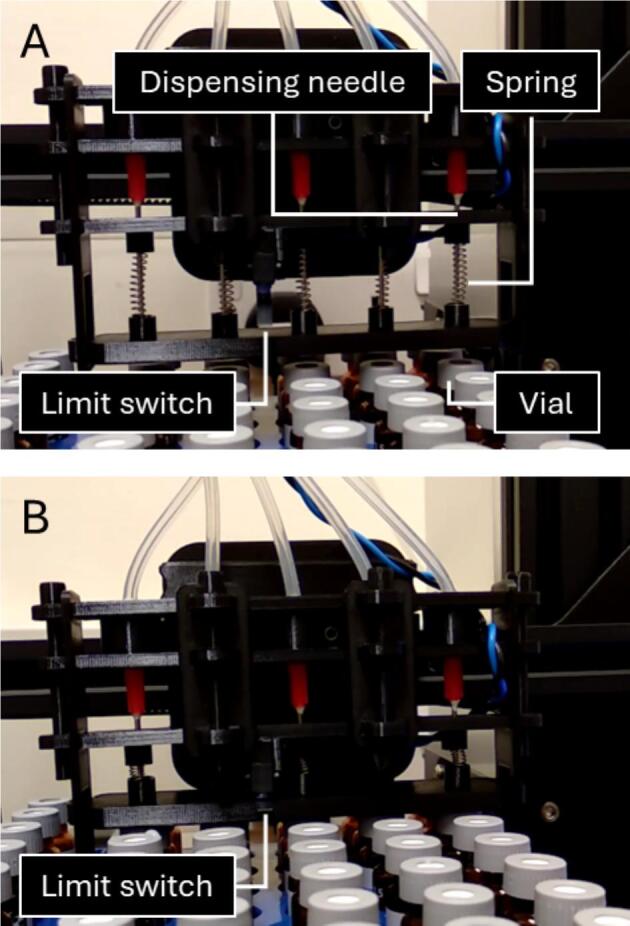
(A) In the 'needles-up' position, the dispensing needles are aligned with the vials. The springs are relaxed and the limit switch is not depressed. The peristaltic pumps are not activated. (B) In the 'needles-down' position, the dispensing needles are inserted into the vials. The springs are compressed and the limit switch is depressed by the pusher. The peristaltic pumps are activated. In normal operation, the dispenser module rises when sampling is complete while the springs push the pusher downwards to aid needle retraction from the vials. The dispenser assembly returns to the ‘needles-up’ position in (A), releasing the limit switch and deactivating the pumps.

Needle-vial alignment is achieved by calibrating the dispenser position along the X- and Y-axes. With the stepper motors enabled, home the gantry using the ‘Autohome’ command from the control panel. Then, raise the needles to the ‘needles-up’ position (Section 6.1.2) and align them vertically with the centre of the vials, using the ‘Move axis’ command from the control panel to calibrate the X- and Y-axis positions. Perform this alignment with all vials in the first row of the vial rack (i.e., 5 on the left and 5 on the right, corresponding to the first and eleventh sampling points). Record the X-axis positions (left and right) and the starting Y-axis position. Then, using the ‘Move axis’ command from the control panel, lower the dispensing needles gradually in 1 mm decrements along the Z-axis, until they penetrate the septa and reach the desired depth inside the vials. In this position, the limit switch should be depressed, the LED illuminated and all pumps activated. Record the Z-axis position as the ‘needles-down’ position (Fig. 7).

The recorded axis positions above may vary slightly between builds, depending on such factors as the height of the build plate and the dimensional accuracy of the gantry and 3D-printed parts. In our build, the recorded values were:

• X-axis position, left (sampling points 1–10): 14.0  mm
• X-axis position, right (sampling points 11–20): 118.5  mm
• Starting Y-axis position: 63.0  mm
• Y-axis increment: 19.0 mm
• ‘Needles-up’ Z-axis position: 27.0  mm
• ‘Needles-down’ Z-axis position: 6.0  mm

**Fig. 8:**
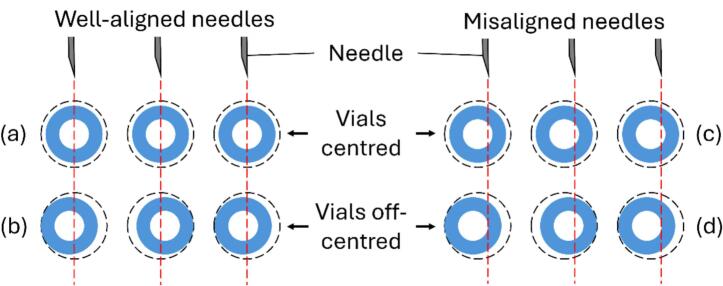
Schematic diagram depicting different scenarios when aligning the needles (red dotted vertical lines) with the pockets in the vial rack (dotted circles), considering that the vial caps (thick blue circles) may be off-centred relative to the pockets. The septa are denoted by the white middle circles surrounded by the thick blue circles. In scenario (a), the needles are well-aligned and the vials are centred, so the needles will penetrate the septa successfully. In (b), the needles are aligned but the vials are off-centred. The large septa provide enough tolerance to ensure the needles can still penetrate successfully. In (c), the needles are misaligned but the vials are centred. The large septa still provide sufficient tolerance for successful needle penetration. In (d), the needles are misaligned and the vials are off-centred. Some needles will not penetrate the septa but will crash into the hard vial caps (thick blue circles). Given that the vials are most likely off-centred, scenarios (a) and (c) are unrealistic. The calibration procedure aims to achieve scenario (b) but avoid scenario (d). (For interpretation of the references to colour in this figure legend, the reader is referred to the web version of this article.)

Despite the calibration procedure above, the needle alignment may still vary slightly between rows of autosampler vials. The dispensing needles have been designed such that they are spaced according to the distance between adjacent pockets of the vial rack. However, the pockets are approximately 1 mm larger in diameter than the vials, so the vials may be slightly off-centre when positioned in the pockets. Nonetheless, the vial septum is large enough that there is sufficient tolerance for this variability provided that the needles are generally aligned centrally with the pockets (Fig. 8). If necessary, the needle-vial alignment may be finetuned as described below.

In our initial setup, misalignment along the X-axis caused some needles to crash into the hard plastic cap, missing the septum altogether. This caused the Z-axis to slip, in a manner similar to Z-axis binding in 3D printing, and the dispensing needles to drift from the calibrated positions. It also has the potential to break the dispenser assembly, although we have not experienced this. The impact of needle-vial misalignment along the X-axis can be amplified because different vials in the same row may lean in opposite directions (i.e., some towards the left, others towards the right), thus significantly altering the spacing between the vials. To finetune the needle-vial alignment, we manually positioned the dispensing needles into each vial across the vial rack and visually determined the deviation of each needle along the X-axis from the centre of the vial. Most needles deviated slightly (40 %) or significantly (42 %) to the right, with only 18 % of all needles penetrating the septa centrally (Table 3). We corrected this by slightly reducing the X-axis position, attaining 70 % central alignment, with the remaining 30 % only slightly misaligned to the right (29 %) or left (1 %) (Table 4). In this configuration, the dispensing needles entered and exited all vials flawlessly. This highlights the importance of diligent calibration and the benefit of being able to adjust these parameters easily in the OttoMate software.

**Table 3:** Needle-vial alignment in the initial setup, recorded as degree of deviation from the centre relative to the vial: C = centre (18 %), CR = centre-right (40 %), R = right (42 %). The column and row headings correspond to the numbered positions in the vial rack (i.e., A1 = leftmost position in row 1). This procedure was not performed beyond the 10th row of vials because it was already clear that the misalignment needed to be corrected.

	A	B	C	D	E
1	C	CR	CR	CR	C
2	CR	C	C	C	CR
3	CR	C	CR	C	CR
4	R	C	CR	CR	R
5	R	CR	R	C	R
6	R	CR	R	CR	R
7	R	CR	R	R	R
8	R	CR	R	CR	CR
9	R	CR	R	R	R
10	R	CR	R	CR	R

**Table 4:** Needle-vial alignment in the optimised setup, recorded as degree of deviation from the centre relative to the vial: C = centre (70 %), CL = centre-left (1 %), CR = centre-right (29 %). The column and row headings correspond to the numbered positions in the vial rack (i.e., A1 = leftmost position in row 1).

	A	B	C	D	E
1	C	C	C	C	C
2	CR	C	C	CR	C
3	CR	C	C	CR	CR
4	CR	C	CR	CR	C
5	CR	CR	CR	CR	CR
6	CR	CR	CR	C	C
7	CR	C	CR	C	C
8	CR	C	C	C	CR
9	C	C	C	CR	CR
10	CR	C	CR	CR	CR
11	C	C	C	C	C
12	C	C	C	C	C
13	C	C	C	C	C
14	C	C	C	C	C
15	C	C	C	CL	C
16	C	C	C	C	C
17	CR	C	C	C	C
18	C	C	C	C	C
19	C	C	C	C	C
20	CR	C	C	C	CR

### 6.2 Programming

Otto is programmed using plain-text G-codes. Use the OttoMate companion app (https://github.com/ngkengwooi/ottomate) to generate G-codes for Otto. Run parameters, such as the calibrated axis positions and the number of sampling points and the sampling interval, can be customised. The graphical user interface is self-explanatory. OttoMate generates a priming G-code (.gcode) file alongside the G-code file for the sampling and refilling routine. Download and save both files to a microSD card and insert it into the microSD card reader in Otto.

### 6.3 Deployment

#### 6.3.1 Dry run

Before deploying Otto in a FDC experiment, we recommend performing a dry run to ensure it functions as intended when unattended. The G-codes for the dry run should be generated using OttoMate to simulate a sampling-refilling routine for 20 sampling points, with a 1-second sampling duration and a 1-second sampling interval.

#### 6.3.2 Priming the refill line

To deploy Otto in a FDC experiment, a sufficient amount of fresh receptor fluid should be loaded into the syringes of the refiller module. To refill the syringes, install the refill line and remove the check valves. Immerse the distal end (where the check valves have been removed) of the refill line in the receptor fluid and pull back on the plungers to fill the syringes. Be careful not to over-extend the plungers as this could inadvertently remove them from the syringe barrel, spilling the receptor fluid on to the electronics below the vial rack. To remove air bubbles from the syringes, push the plungers to force the air fully out of the refill line and pull back again to fill the syringes with more receptor fluid. Repeat this step as many times as needed to completely purge the syringes and the refill line of air bubbles. Then, with the syringes filled and the plungers fully extended, install the check valves and refill needle to the distal end of the refill line. Place the refill needles in a beaker and run the priming G-code from the microSD card, by executing the “Run from TF” command from the control panel. The X-axis should rise to the pre-set position and push out excess receptor fluid from the refill line into the beaker. The refill line is now primed.

#### 6.3.3 Setting up the Franz diffusion cells

Next, set up the FDCs in the water bath. Fill the receptor chamber with the receptor fluid but do not load the drug formulation in the donor chamber yet. Insert a refill needle and the correct sampling needle, through the needle holder, into the sampling port of each FDC. Accurate needle placement is essential. The sampling needle should terminate within the sampling port, just above the junction with the receptor chamber. The refill needle must be inserted fully into the receptor chamber. Incorrect needle placement, such as shallow insertion of the refill needle or overly deep placement of the sampling needle, can introduce air bubbles or cause airlocks, which can disrupt fluid exchange and compromise sampling accuracy. Each sampling line must also be correctly matched to its corresponding dispensing needle on the Otto platform to ensure that the samples are transferred into the correct vials. The refill needles are interchangeable, as all syringes in the refiller module contain the same receptor fluid. Install the needle lock to the FDC assembly to prevent displacement of the needles. Immerse ∼25  mm of the distal refill line in the water in the water bath for temperature control.

#### 6.3.4 Equilibration

We have found that performing an equilibration run comprising 2–3 sampling and refill cycles greatly improves sample volume consistency and thus data accuracy [6]. The G-code for this can be generated using OttoMate, with the same parameters as the dry run, but for 2–3 sampling points only. This will collect some receptor fluid from the FDCs into the sample vials. Used but clean sample vials may be used for this purpose. If the experimental design allows, this equilibration run may be incorporated into the G-code for the actual experiment. Otherwise, the refill line may need to be re-primed before the experiment.

#### 6.3.5 Start the experiment

Once Otto has been primed and the equilibration run has completed, replace any used autosampler vials with new ones, as appropriate. Then, load the drug formulation in the donor chamber and start the sampling/refilling routine by executing the relevant G-code file from the microSD card, using the “Run from TF” command from the control panel.

## 7 Validation and characterisation

Otto has been extensively tested and validated against key performance parameters, including temperature control, sample volume consistency and data accuracy. The validation experiments have been detailed elsewhere [6] and are summarised below. All reported *p* values were derived from two-tailed paired t-tests at *α* = 0.05.

The receptor fill volume was compared between Otto and manual operation, to ascertain if the in-situ sampling and refill needles may displace a significant amount receptor fluid to affect the results. The receptor fill volume for Otto was 2.247 ± 0.028 mL (mean ± SD, *n* = 5), while manual sampling resulted in a slightly higher fill volume of 2.396 ± 0.134 mL. The difference in fill volume between Otto and manual sampling was marginal (*p* = 0.049; paired two-tailed *t*-test). The residual volume, or the amount of fluid retained in the sampling tube after sampling, was also minimal (mean = 1.2  µL, SD = 0.9 µL, *n* = 5), equating to about 0.5 % of the total sample volume [6].

Temperature control was effectively maintained by immersing a 25  cm segment of the refill line (the temperature control loop) in the water bath. The temperature of the refill line past the temperature control loop, immediately before entering the FDCs, was within 0.5 °C of the water surface temperature [6]. Thus, the temperature control loop effectively pre-warmed the fresh receptor fluid before filling it into the FDCs.

**Fig. 9:**
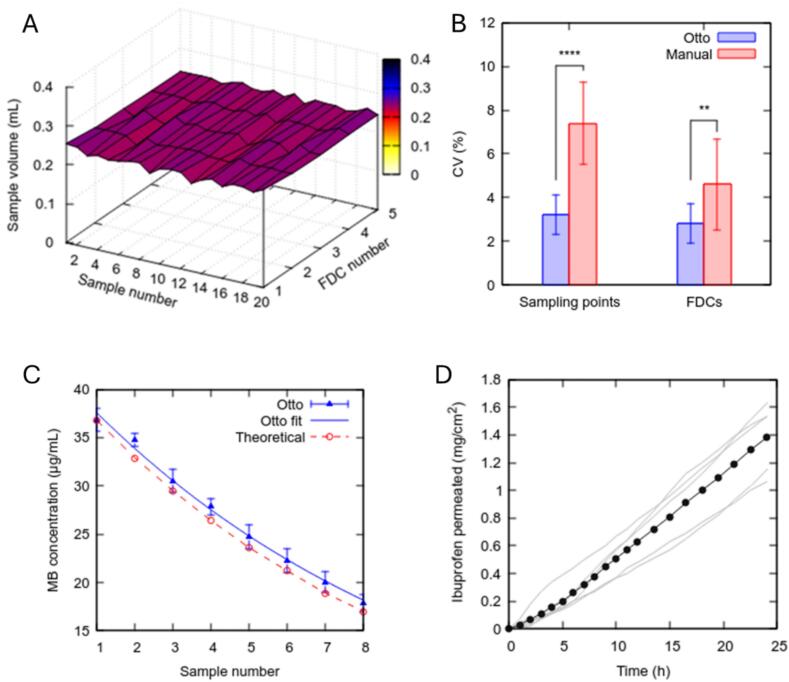
Otto's validation results. (A) Otto’s sample volume consistency. The flat surface plot signifies consistent volume was achieved across sampling points and across FDCs. (B) Otto’s sample volume was less variable than manual operation both across sampling points and across FDCs (****p < 0.0001; **p < 0.01). (C) Empirically determined MB concentration in samples extracted by Otto closely tracked the theoretical concentration in the accuracy assessment, using the geometric dilution approach, demonstrating high data accuracy. (D) Ibuprofen permeation profile produced using Otto. The solid line is the mean of 5 replicates, shown as grey lines. Images cropped from Fig. 4, Fig. 5, Fig. 6, Fig. 7 of Chan et al. [6], used under CC BY 4.0.

Otto demonstrated superior sample volume consistency compared to manual operation (Fig. 9A–B). The mean sample volume for Otto was 241.5 µL (SD = 7.8 µL, *n* = 100), with a coefficient of variation (CV) of 3.2 %. In comparison, manual sampling had a mean of 239.5 µL (SD = 18.0 µL, *n* = 30) and a higher CV of 7.5 %. Otto's sample volume consistency was significantly better both across different sampling points (*p* < 0.0001) and across FDCs (*p* < 0.01; unpaired two-tailed *t*-test). Early tests with Otto showed variability in the first two samples, but this was resolved by performing an equilibration cycle before each run, resulting in consistent volumes similar to manual sampling [6].

Data accuracy was determined using the spike-and-recovery and geometric dilution. In spike-and-recovery, the FDC receptor chamber was initially filled with deionised water and refilled with a known concentration of methylene blue (MB, as a tracer molecule) solution at every sampling point. In geometric dilution, the FDC receptor chamber was initially filled with a MB solution of known concentration and refilled with deionised water at every sampling point. In both cases, the theoretical amount of MB in each sample may be calculated and compared with the empirical values. In the spike-and-recovery test, Otto’s data accuracy was comparable to manual operation, although both fell slightly short of the theoretical values due to technical limitations of the laboratory methods (i.e., inherent errors associated with multiple pipetting steps and spectrophotometry). Using high-performance liquid chromatography with no additional pipetting, Otto’s empirical concentrations closely tracked the theoretical concentrations in the geometric dilution test, confirming its high accuracy (Fig. 9C).

In terms of in-use performance, Otto demonstrated the anticipated near-constant ibuprofen permeation rate over 24 h (Fig. 9D), similar to manual operation when the full media replacement sampling regime (i.e., replacing the entire receptor volume with fresh media) was used. In contrast, the manual aliquot sampling regime (i.e., sampling manually from the sampling port only, similar to Otto) showed reduced flux between 4 and 21 h, during which manual sampling was paused to avoid out-of-hours working. With aliquot sampling, ibuprofen was allowed to accumulate in the receptor chamber more rapidly, so it was more important to sample frequently to maintain a steep concentration gradient and thus a constant permeation rate. The results suggested that the sampling gap had unduly suppressed ibuprofen permeation, by allowing ibuprofen to accumulate excessively in the receptor chamber, leading to an underestimation of drug absorption. Despite implementing aliquot sampling, Otto maintained a steady permeation profile without such fluctuations, as it continued to operate unattended outside normal working hours. Thus, Otto offered more reliable permeation data compared to manual operation. We have deployed Otto in several experiments, including in a published study [14], and it has performed reliably without issues.

## Ethics statements

This work did not require ethics approval. It did not involve any human subject or animal experimentation.

## CRediT authorship contribution statement

**Keng Wooi Ng:** Writing – review & editing, Writing – original draft, Visualization, Validation, Supervision, Software, Methodology, Investigation, Funding acquisition, Formal analysis, Conceptualization. **Liam Archbold:** Writing – review & editing, Investigation, Formal analysis. **Wing Man Lau:** Writing – review & editing, Supervision, Funding acquisition.

## Declaration of competing interest

The authors declare that they have no known competing financial interests or personal relationships that could have appeared to influence the work reported in this paper.
