# Supplementary material for: Building Otto: An open-source Franz diffusion cell autosampler for automating in vitro skin permeation studies
Source: HardwareX. 2025 Dec 21;25:e00735. doi: 10.1016/j.ohx.2025.e00735 (PMC12808608; doi:10.1016/j.ohx.2025.e00735)
Supplement: Supplementary Data 1 [file mmc1.pdf]

List of 3D-printable parts for Otto, the Franz diffusion cell autosampler

Keng Wooi Ng
keng.ng@newcastle.ac.uk
ORCID: 0000-0003-2541-4763
Web: https://linktr.ee/ngkengwooi

28 October 2025

Parts are not shown to scale. Number in parentheses indicates number of copies to print.

Part names correspond to CAD file names, e.g. the CAD file for sampler-backplate is sampler-backplate.fcstd.

Unless otherwise indicated, parts were printed in PETG with a 0.4 mm nozzle, at 0.20 mm layer height, 15% gyroid infill.

Related publications:

1. Ng KW. Design files for Otto, the Franz diffusion cell autosampling robot. Mendeley Data, V1, 2025. https://doi.org/10.17632/cvc9vxjgn9.1
2. Chan HKY, Archbold L, Lau WM, Ng KW. Validating Otto: a Franz diffusion cell autosampler to automate in vitro permeation studies, Journal of Pharmaceutical Sciences, 2025:103837. https://doi.org/10.1016/j.xphs.2025.103837

Sampler module

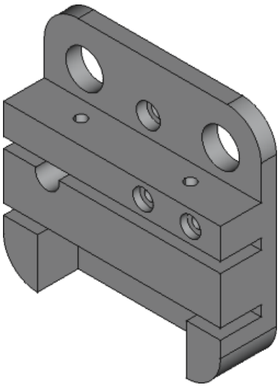 sampler-backplate (1)	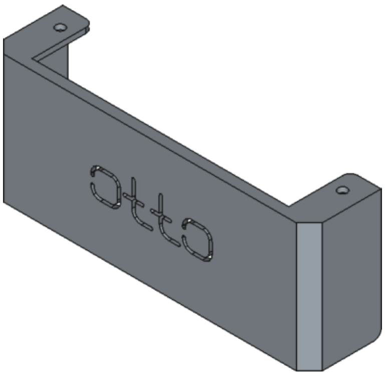 sampler-front-cover (1)	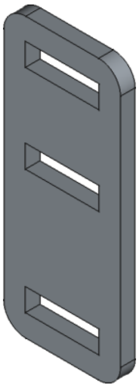 sampler-front-panel (2)
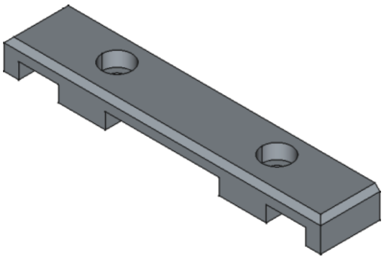 sampler-ledge-top (1)	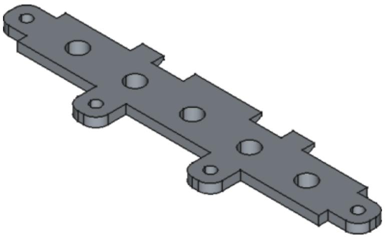 sampler-lid (1)	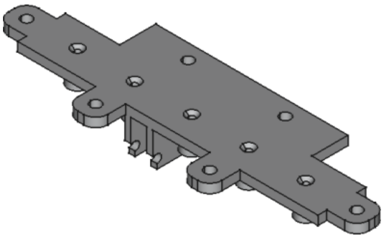 sampler-needle-aligner (1)
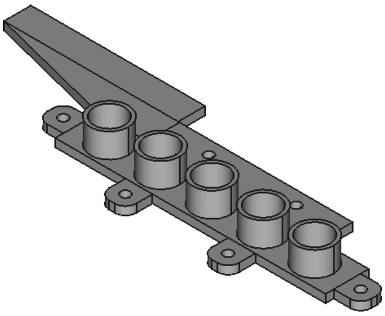 sampler-needle-holder (1)	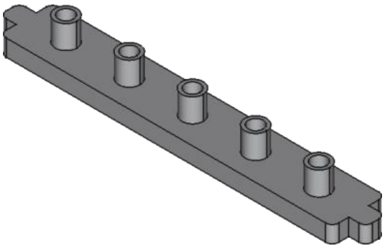 sampler-pusher (1)<br>Print at 100% infill	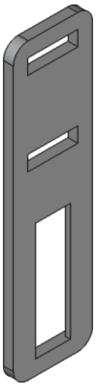 sampler-side-panel (2)
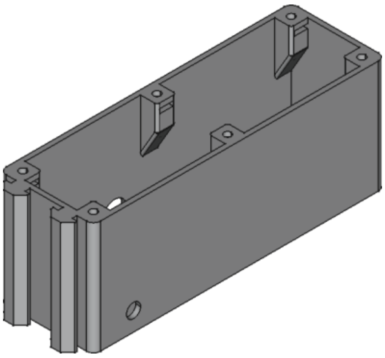 electronic-enclosure (1)	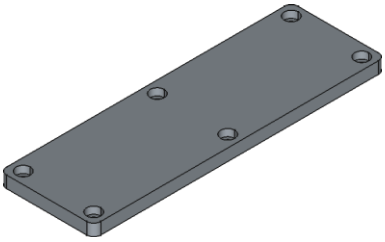 electronic-enclosure-lid (1)	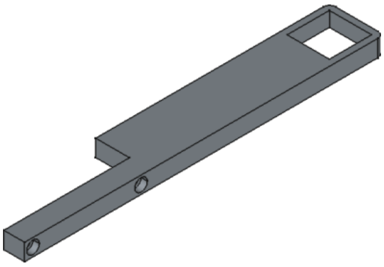 pump-bracket-hangdown (1)

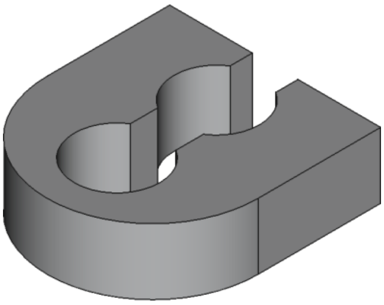 pump-duplex-tubing-clip (6)	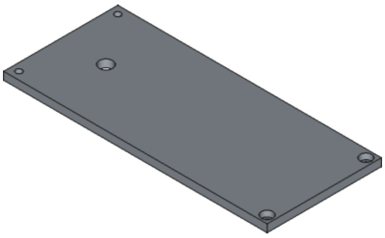 pump-panel-back (1)	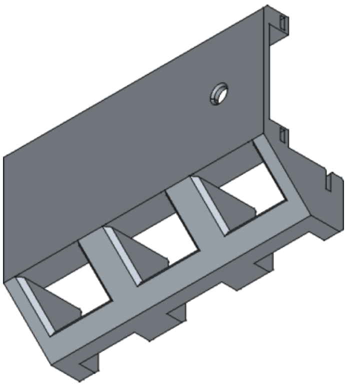 pump-panel-front (1)

Refiller module

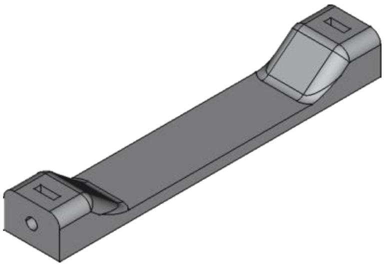<br>refiller-bracket-bottom (1)	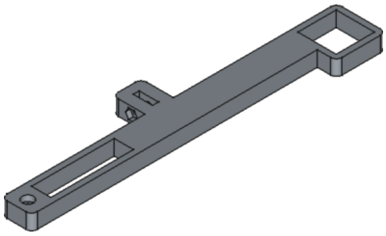<br>refiller-bracket-hangdown (Right bracket provided, mirror print for left bracket, 1 each)	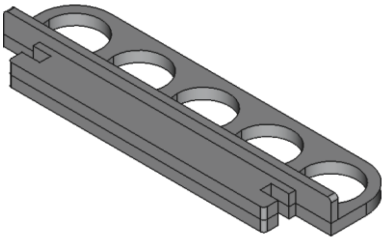<br>refiller-bracket-middle (1)
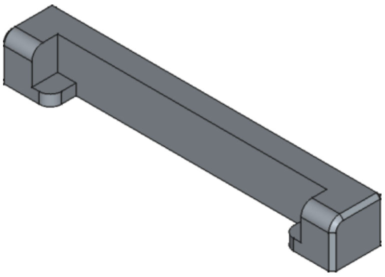<br>refiller-bracket-middle-lock (1)	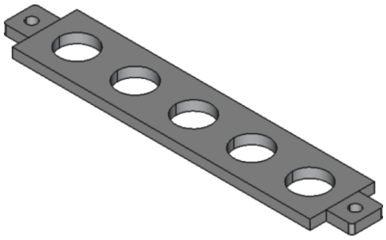<br>refiller-bracket-top (1)	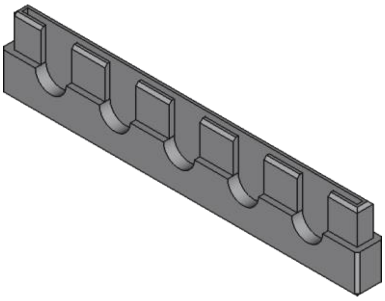<br>refiller-plunger-lock-a (1)
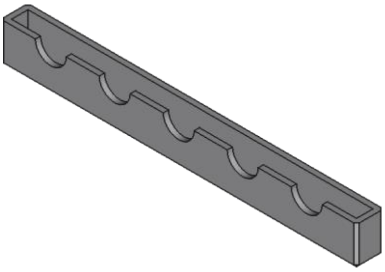<br>refiller-plunger-lock-b (1)		

Vial rack module

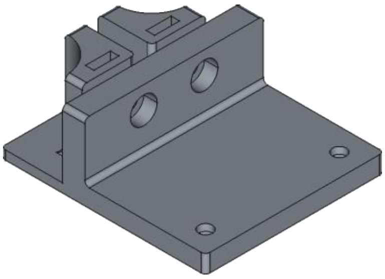<br>rack-front-bracket-a (1)	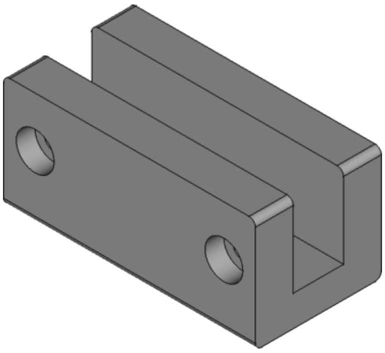<br>rack-front-bracket-b (1)	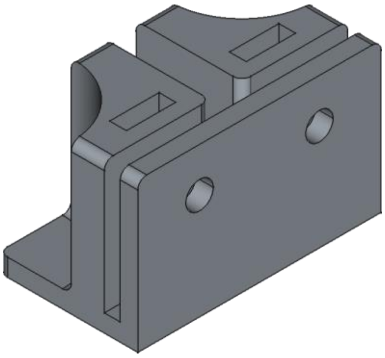<br>rack-rear-bracket-a (1)
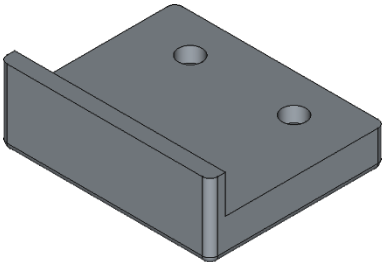<br>rack-rear-bracket-b (1)	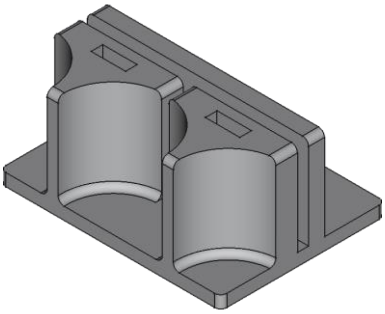<br>rack-side-bracket-a (4)	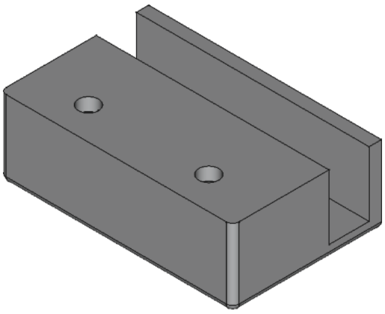<br>rack-side-bracket-b (4)

Franz diffusion cell (FDC) module

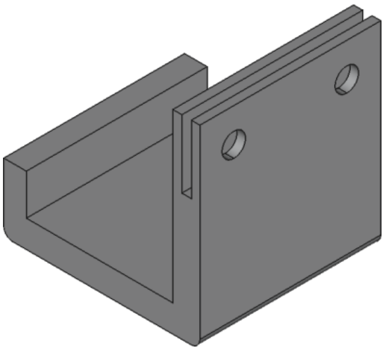 fdc-bracket (4)	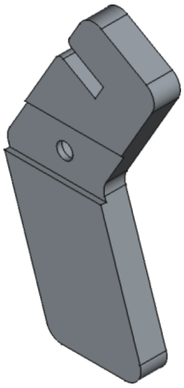 fdc-leg<br>(Right leg provided, mirror print for left leg, 5 each)	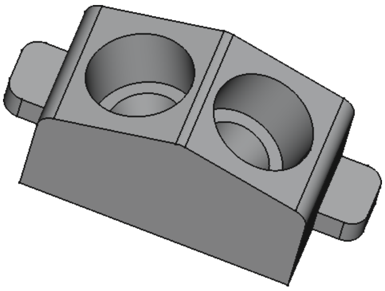 fdc-needle-holder (5)
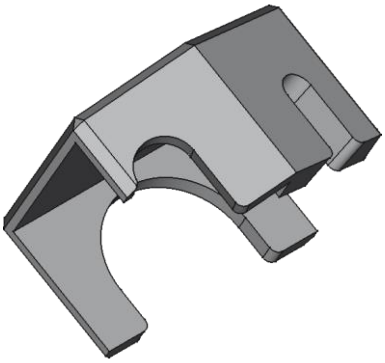 fdc-needle-lock (5)	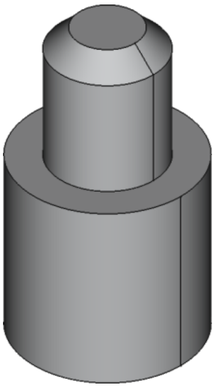 fdc-pin (8)	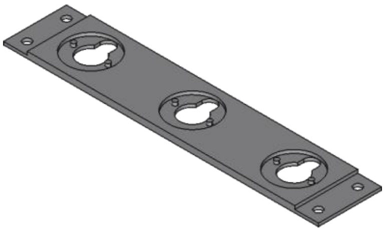 fdc-plate (2)
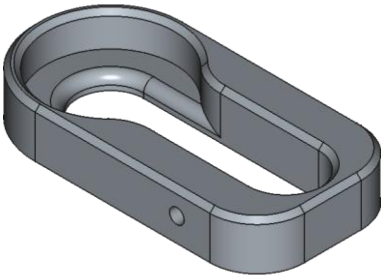 fdc-seat (5)	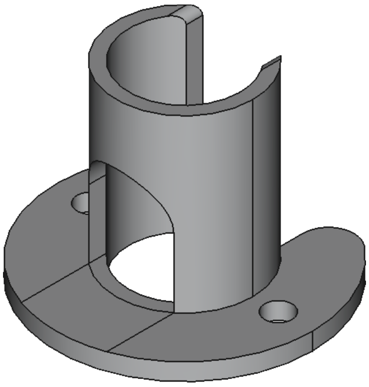 fdc-stand (5)	
